# In vivo toxicity evaluation of a polyoxotungstate nanocluster as a promising contrast agent for computed tomography

**DOI:** 10.1038/s41598-023-36317-8

**Published:** 2023-06-05

**Authors:** Marko Stojanović, Jovana Lalatović, Aleksandra Milosavljević, Nada Savić, Charlotte Simms, Branimir Radosavljević, Mila Ćetković, Tamara Kravić Stevović, Davor Mrda, Mirjana B. Čolović, Tatjana N. Parac-Vogt, Danijela Krstić

**Affiliations:** 1 Department of Pharmacology, Clinical Pharmacology and Toxicology, Faculty of Medicine, University of Belgrade, Belgrade, Serbia; 2 Department of Radiology, University Hospital Medical Center Bežanijska Kosa, Belgrade, Serbia; 3 Institute of Histology and Embryology, Faculty of Medicine, University of Belgrade, Belgrade, Serbia; 4 Department of Chemistry, KU Leuven, Celestijnenlaan 200F, 3001 Leuven, Belgium; 5 Institute of Medical Chemistry, Faculty of Medicine, University of Belgrade, Belgrade, Serbia; 6 “Vinča” Institute of Nuclear Sciences-National Institute of the Republic of Serbia, University of Belgrade, Belgrade, Serbia

**Keywords:** Medicinal chemistry, Toxicology, Biomaterials, Metals

## Abstract

In this study, we demonstrate for the first time, that a discrete metal-oxo cluster α-/β-K_6_P_2_W_18_O_62_ (WD-POM) exhibits superior performance as a computed tomography (CT) contrast agent, in comparison to the standard contrast agent iohexol. A toxicity evaluation of WD-POM was performed according to standard toxicological protocols using *Wistar albino* rats. The maximum tolerable dose (MTD) of 2000 mg/kg was initially determined after oral WD-POM application. The acute intravenous toxicity of single WD-POM doses (1/3, 1/5, and 1/10 MTD), which are at least fifty times higher than the typically used dose (0.015 mmol W kg^−1^) of tungsten-based contrast agents, was evaluated for 14 days. The results of arterial blood gas analysis, CO-oximetry status, electrolyte and lactate levels for 1/10 MTD group (80% survival rate) indicated the mixed respiratory and metabolic acidosis. The highest deposition of WD-POM (0.6 ppm tungsten) was found in the kidney, followed by liver (0.15 ppm tungsten), for which the histological analysis revealed morphological irregularities, although the renal function parameters (creatinine and BUN levels) were within the physiological range. This study is the first and important step in evaluating side effects of polyoxometalate nanoclusters, which in recent years have shown a large potential as therapeutics and contrast agents.

## Introduction

Computed tomography (CT) is a versatile and powerful imaging technique which is nowadays used in many applications in (bio)medical and nonmedical fields. CT plays a prominent role in diagnostic medicine, as a noninvasive technique which can provide detailed 3D images of the body. Additionally, the use of contrast agents enables better visualization of blood vessels, as well as of the clinical features and anatomic details of the investigated regions of the body. Also, pathological lesions are visualized differently on CT scans when contrast agents are applied^1–3^. High-resolution CT images, as well as the widespread availability of clinical CT scanners, are some of the main reasons why CT is becoming an imaging technique of choice in clinics and hospitals around the world.

The currently used contrast agents for CT are mainly derivatives of a 2,4,6-triiodinated benzene ring and are indispensable in the radiology practice, for both diagnostic and therapeutic purposes. Although these iodinated contrast media are well tolerated, adverse reactions, from minor to severe-life threatening, may occur after injection of a contrast agent. Moreover, contrast media-induced acute kidney injury (AKI) has been recognized as the third leading cause of hospital-acquired AKI^4,5^. Furthermore, the application of iodinated CT contrast poses a high risk for patients with thyroid disease, as well as for renally impaired patients and those hypersensitive to iodinated agents. Thus, the documented severe reactions to iodinated contrast media, including anaphylaxis, angioedema, and bronchospasm are considered as absolute contraindications for their use. Additionally, the essential disadvantages of iodinated contrast agents are rapid clearance, inefficient targetability, and poor sensitivity. Due to the above limitations, the search for low-cost, nontoxic CT contrast agents with maximum imaging capabilities and minimal dose requirements is an ongoing challenge^6–8^.

Recent studies demonstrated that metallic nanoparticles are suitable candidates for the development of new generation CT contrast agents since metals have high X-ray attenuation and high density^9^. Zirconium-substituted Keggin polyoxometalate (POM) was reported as a non-destructive contrast agent that allows the development of the intact placenta to be visualized using microCT^10^. Moreover, some Wells–Dawson polyoxotungstates such as parent, α-/β-K_6_P_2_W_18_O_62_ (WD-POM), the 1:2 hafnium(IV)-substituted, K_16_[Hf(α_2_-P_2_W_17_O_61_)_2_]·19H_2_O (Hf-WD POM), monolacunary, α2-K_10_P_2_W_17_O_61_^.^20H_2_O (Mono-WD POM), and trilacunary, K_16_[Hf(α_2_-P_2_W_17_O_61_)_2_]·19H_2_O (Tri-WD POM) have been reported as promising contrast-enhancing staining agents to visualize kidney, as well as bone and its marrow vascularization and adiposity using microCT^11,12^.

In this study in vitro CT imaging properties of WD-POM as a representative of promising CT contrast agent class based on POMs were investigated by recording x-ray attenuation phantom images and compared with iohexol, a most commonly used contrast agent in clinical applications. The stronger x-ray attenuation of WD-POM compared to iohexol (at the same W/I concentration) demonstrated in this work supports the potential use of POM-based compounds as CT contrast agents. Since toxicity and clinical safety have a major impact on drug development success, the further aim of our study was to evaluate in vivo toxic effects of WD-POM as a representative of promising CT contrast agent class based on POMs, using *Wistar albino* rats as an experimental model. In addition, tissue distribution of WD-POM was evaluated two weeks after intravenous application of 1/10 of the maximum tolerable dose (MTD) by measuring tungsten concentrations in different tissue (liver, kidneys, and brain) and urine samples, using Inductively Coupled Plasma-Optical Emission Spectrometry (ICP-OES). These results also provide important insight into the potential toxic effects of Wells–Dawson polyoxotungstates and POMs in general, as they have been frequently investigated as promising antitumor, antiviral, and antibacterial agents^13–17^.

## Results

### In vitro CT imaging

**Figure 1:**
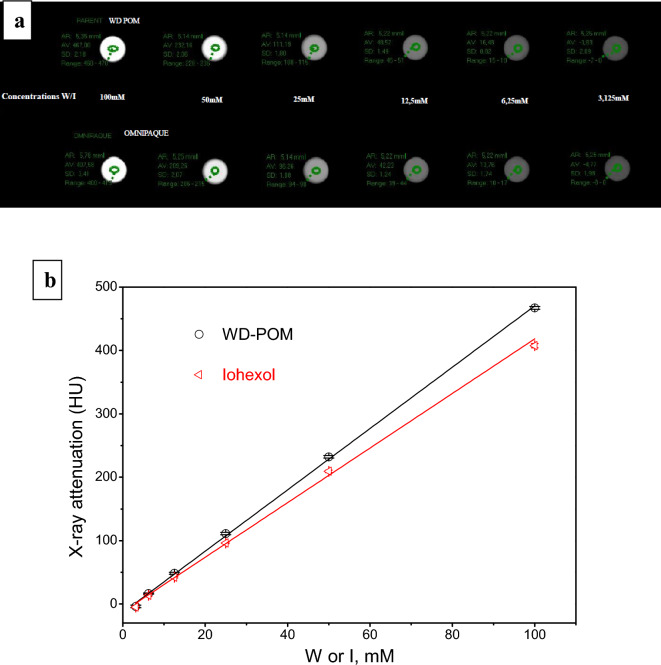
In vitro CT phantom images (**a**) and the dependence of X-ray attenuation (expressed in the Hounsfield units (HU)) on W/I concentration (**b**), for WD-POM and iohexol.

The results of the x-ray attenuation (expressed in the Hounsfield units (HU)), obtained for aqueous WD-POM/iohexol solutions at the x-ray source voltage of 80 kV, as a function of the same W/I concentrations (3.125–100 mM) and the corresponding in vitro CT phantom images are presented in Fig. 1. The experimental plots fitted excellently a linear function for both WD-POM and iohexol (R^2^ values were 0.99951 and 0.99792, respectively). Apparently, the observed HU values for WD-POM of interest were significantly higher than those for the reference iohexol, at the same W/I concentrations. Accordingly, a significantly higher slope of the linear function was obtained for WD-POM compared to iohexol (4.83708 vs. 4.30594). These results indicated the superior contrast performance of WD-POM for CT imaging related to the standard contrast agent that has been most frequently used in clinical practice.

### Mortality and physical changes

**Figure 2:**
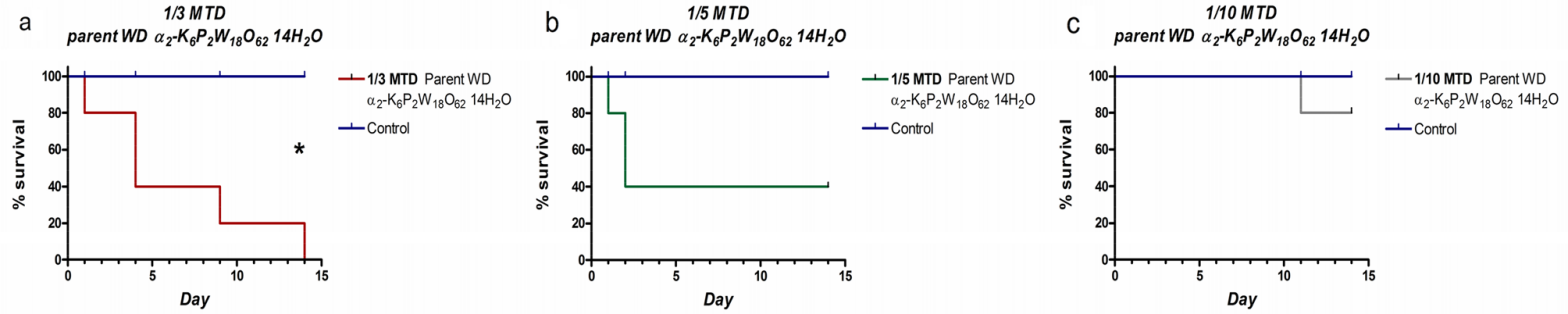
Kaplan–Meier survival analysis for the 1/3 MTD (**a**), 1/5 MTD (**b**), and 1/10 MTD (**c**) groups for the 14 days follow-up period in comparison with the control group. *p < 0.05.

Kaplan–Meier survival analysis was used for 14-day monitoring rat mortality rate in four groups: (1) control (saline), (2) 1/3 MTD (666.7 mg/kg WD-POM), (3) 1/5 MTD (400 mg/kg WD-POM), and (4) 1/10 MTD (200 mg/kg WD-POM) and presented in Fig. 2. In the 1/3 MTD group, the survival rate was 0% (0/5) after 14 days follow-up, which is significantly lower in comparison to the control group (p = 0.02) (Fig. 2a). As the WD-POM dose was reduced, the survival rate increased resulting in 40% for 1/5 MTD (2/5) (Fig. 2b), whereas the highest survival rate (80% (4/5)) was observed for 1/10 MTD group (Fig. 2c).

**Figure 3:**
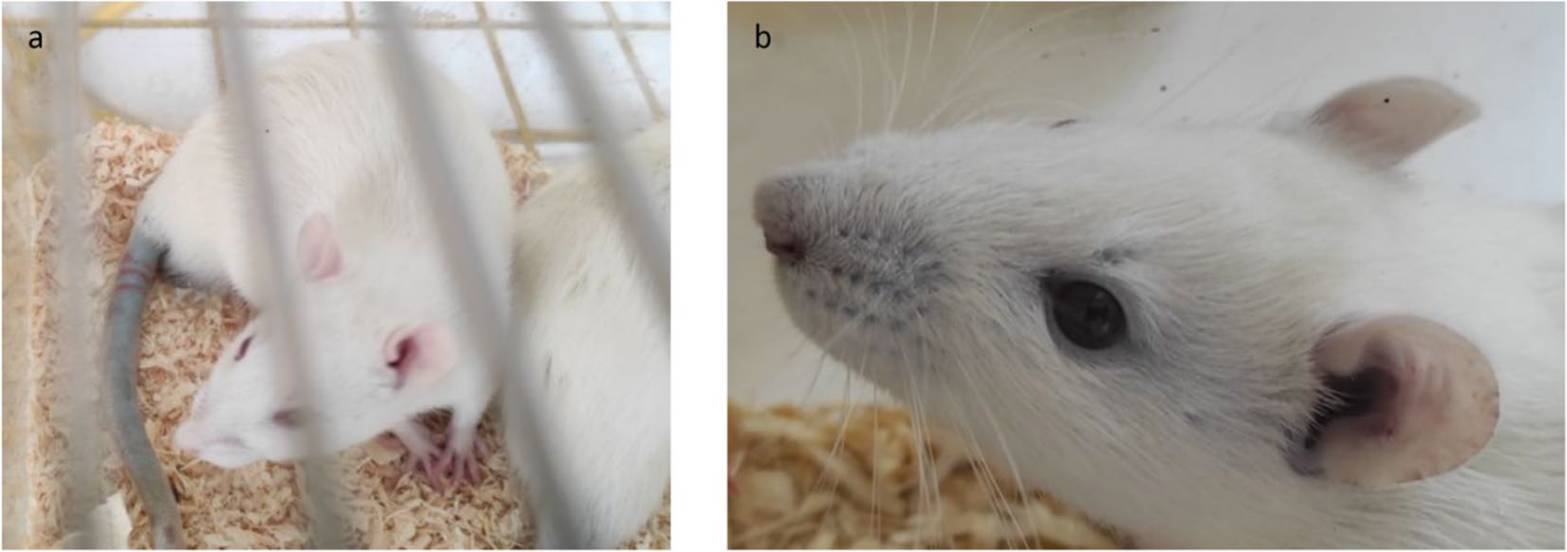
The blue discoloration of the rat tail 24 h after WD-POM administration (**a**), the change in the skin and eyeball color 14 days after WD-POM administration (**b**).

The 14-day observation of the physical changes of the treated animals showed that in the first 24 h post-WD-POM application, in all three experimental groups, the animal tail changed color to dark blue (Fig. 3a). At the end of the follow-up period, the skin, visible mucosa, and eyeball color became blue (Fig. 3b) in each survived animal, regardless of the studied group. The appearance of dark blue color suggests that the reduction of WD-POM took place, as it has been previously reported that WD undergo color change from colorless to deep blue upon reduction of tungsten atoms in the structure of WD^18^.

### Laboratory blood analysis

Arterial blood gas analysis, CO-oximetry status, as well as the levels of electrolytes, glucose, lactate, creatinine, and BUN were measured 14 days after the treatment in the control and 1/10 MTD group (% survival was 80).

The results of arterial blood gas analysis (Table 1) demonstrated that after 14-day follow-up period the pH levels were low in both control and 1/10 MTD group, indicating the presence of acidosis, with no statistical difference between the groups (p > 0.05). The arterial pCO_2_ and HCO_3_^−^ levels were significantly higher in 1/10 MTD group (p < 0.01). The pCO_2_ value in 1/10 MTD was above the normal range, whereas HCO_3_^−^ concentration in the control group was below the normal range. The values of blood base excess were significantly different between the two groups (p < 0.05), and both groups had values under the lower limit of the normal range. The anion gap was statistically significantly higher in the control group (p < 0.05), with the control group values above the upper limit of the normal range.

**Table 1:** The results of arterial blood gas analysis of control and animals treated with 1/10 MTD.

Analyte	Units	Control (n = 5)	1/10 MTD (n = 4)	p-value	Normal range
pH	/	7.11 ± 0.09	7.17 ± 0.04	0.6691	7.3–7.4
pCO_2_	kPa	5.38 ± 1.04	8.35 ± 1.17	0.0050**	4.66–5.99
HCO_3_^−^	mmol/l	17.56 ± 3.53	25.71 ± 2.33	0.0055**	21–28
Base excess of blood	mmol/l	− 8.79 ± 4.18	− 3.26 ± 1.80	0.0435*	− 2 to + 3
Anion gap	mmol/l	18.56 ± 4.12	13.06 ± 0.72	0.0354*	8.00–16.00

Values are expressed as mean ± SD; *p < 0.05; **p < 0.01.

The CO-oximetry status of control animals and animals from the 1/10 MTD group is presented in Table 2. The statistically significant difference between the control and 1/10 MTD group was noted for the values of deoxyhemoglobin (p = 0.0324) and oxygen saturation (p = 0.012). The oxygen saturation level in the 1/10 MTD group was below the lower limit of the normal range. Moreover, in this group deoxyhemoglobin level was above the upper limit of the normal range. Interestingly, no statistically significant difference was observed in the level of oxyhemoglobin between the two groups, although the trend of reduction can be seen in the 1/10 MTD group. Additionally, oxyhemoglobin levels were below the lower limit of the normal range in this group.

**Table 2:** The CO-oximetry values in control and 1/10 MTD group of animals.

Analyte	Units	Control (n = 5)	1/10 MTD (n = 4)	p value	Normal range
Total hemoglobin	g/l	166.50 ± 6.84	176.80 ± 11.09	0.1301	140–178
Oxyhemoglobin	%	97.07 ± 0.34	75.13 ± 23.50	0.0616	94–97
Carboxyhemoglobin	%	0.42 ± 0.05	0.45 ± 0.19	0.7406	0.0–1.5
Methemoglobin	%	0.99 ± 0.31	0.70 ± 0.16	0.1327	0.0–1.5
Deoxyhemoglobin	%	0.54 ± 0.074	19.65 ± 16.35	0.0324*	0.0–5.0
Oxygen saturation	%	99.5 ± 0.35	71.44 ± 23.63	0.0120*	95–98
Oxygen content of hemoglobin	vol%	23.57 ± 0.93	18.78 ± 5.48	0.0915	18–24
Oxygen capacity of hemoglobin	vol%	22.81 ± 0.94	24.38 ± 1.62	0.1101	18–25

Values are expressed as mean ± SD; *p < 0.05.

**Figure 4:**
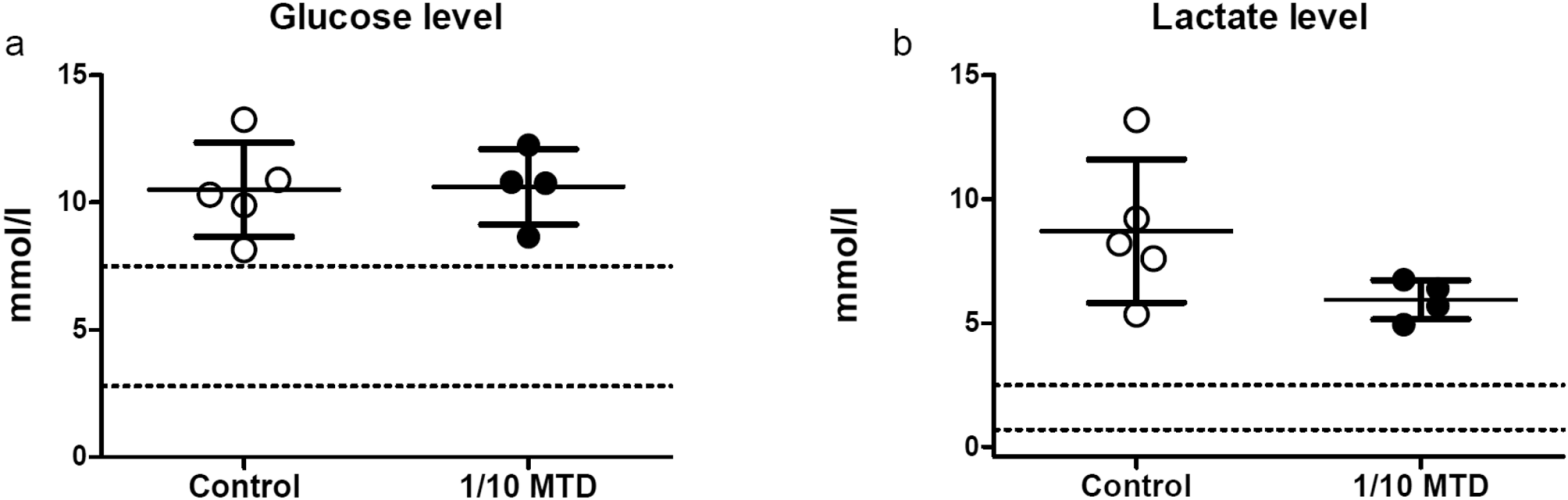
Scatter plot showing the blood glucose level (**a**) and lactate level (**b**). The results are presented as mean ± SD. Normal range is marked with dash lines.

The blood glucose levels in the control group and 1/10 MTD were 10.50 ± 0.83 mmol/l and 10.61 ± 0.74 mmol/l, respectively. The statistical difference between the groups was not obtained and glucose levels in both groups were noticeably above the normal blood glucose range (2.8–7.5 mmol/l). The lactic acid levels were not statistically significantly different between the two groups, with levels of 8.71 ± 1.29 mmol/l in the control, and 5.95 ± 0.40 mmol/l in 1/10 MTD group. Both values were above the upper limit of the normal range (0.7–2.5 mmol/l). The blood glucose and lactate levels are presented in Fig. 4.

The concentration of electrolytes Na^+^, K^+^, Cl^−^, Ca^2+^, Mg^2+^, Ca^2+^, and Mg^2+^ normalized to pH 7.4 are given in Table 3. Generally, there is no statistical difference in the values of these electrolytes between the groups, nor do the values significantly deviate from the normal range. The only difference between the control and 1/10 MTD group was for the potassium values which were statistically significantly lower in the 1/10 MTD group (p = 0.0204), yet in the normal range in both groups.

**Table 3:** Arterial blood electrolyte concentrations in control and 1/10 MTD group.

Analyte	Units	Control (n = 5)	1/10MTD (n = 4)	p value	Normal range
Na^+^	mmol/l	136.00 ± 0.80	138.9 ± 2.08	0.0549	136–146
K^+^	mmol/l	5.14 ± 0.28	4.11 ± 0.34	0.0204*	3.5–5.2
Cl^−^	mmol/l	104.90 ± 1.45	104.30 ± 0.60	0.4283	98–106
Ca^2+^	mmol/l	1.24 ± 0.04	1.3 ± 0.05	0.0658	1.09–1.30
Mg^2+^	mmol/l	0.64 ± 0.10	0.74 ± 0.09	0.1755	0.45–0.65
Ionized calcium normalized to pH 7.4 (nCa)	mmol/l	1.14 ± 0.05	1.30 ± 0.24	0.1901	1.09–1.30
Ionized magnesium normalized to pH 7.4 (nMg)	mmol/l	0.58 ± 0.06	0.66 ± 0.07	0.1165	0.45–0.6

Values are expressed as mean ± SD; *p < 0.05.

**Figure 5:**
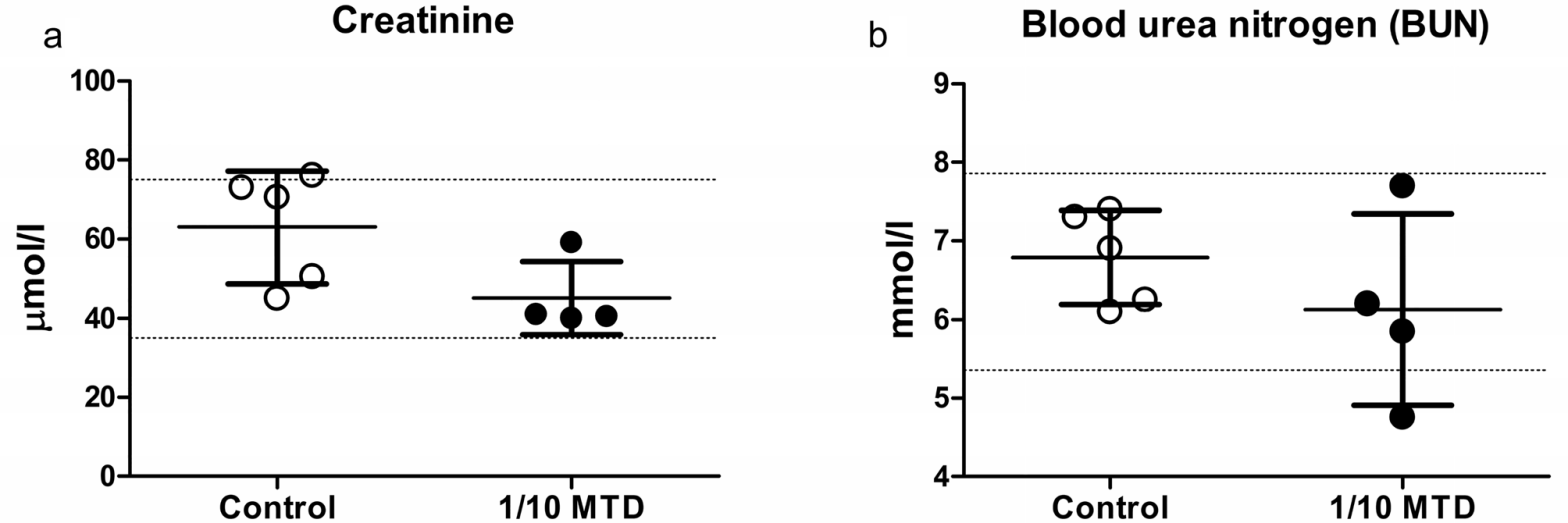
Arterial blood creatinine (**a**) and blood urea nitrogen (BUN) (**b**) values in the control and 1/10 MTD group. The results are presented as mean ± SD. Normal range is marked with dash lines.

The arterial blood creatinine (63 ± 6.35 µmol/l vs. 45.13 ± 4.63 µmol/l; p = 0.9242) and BUN (6.79 ± 0.27 mmol/l vs. 6.13 ± 0.61 mmol/l p = 0.3147) values were not statistically significantly different in the two groups of animals. The obtained mean values of these two parameters, for both groups, were in the normal range. The arterial blood creatinine and BUN values are presented in Fig. 5.

### Histopathological evaluation of WD-POM—induced renal toxicity

**Figure 6:**
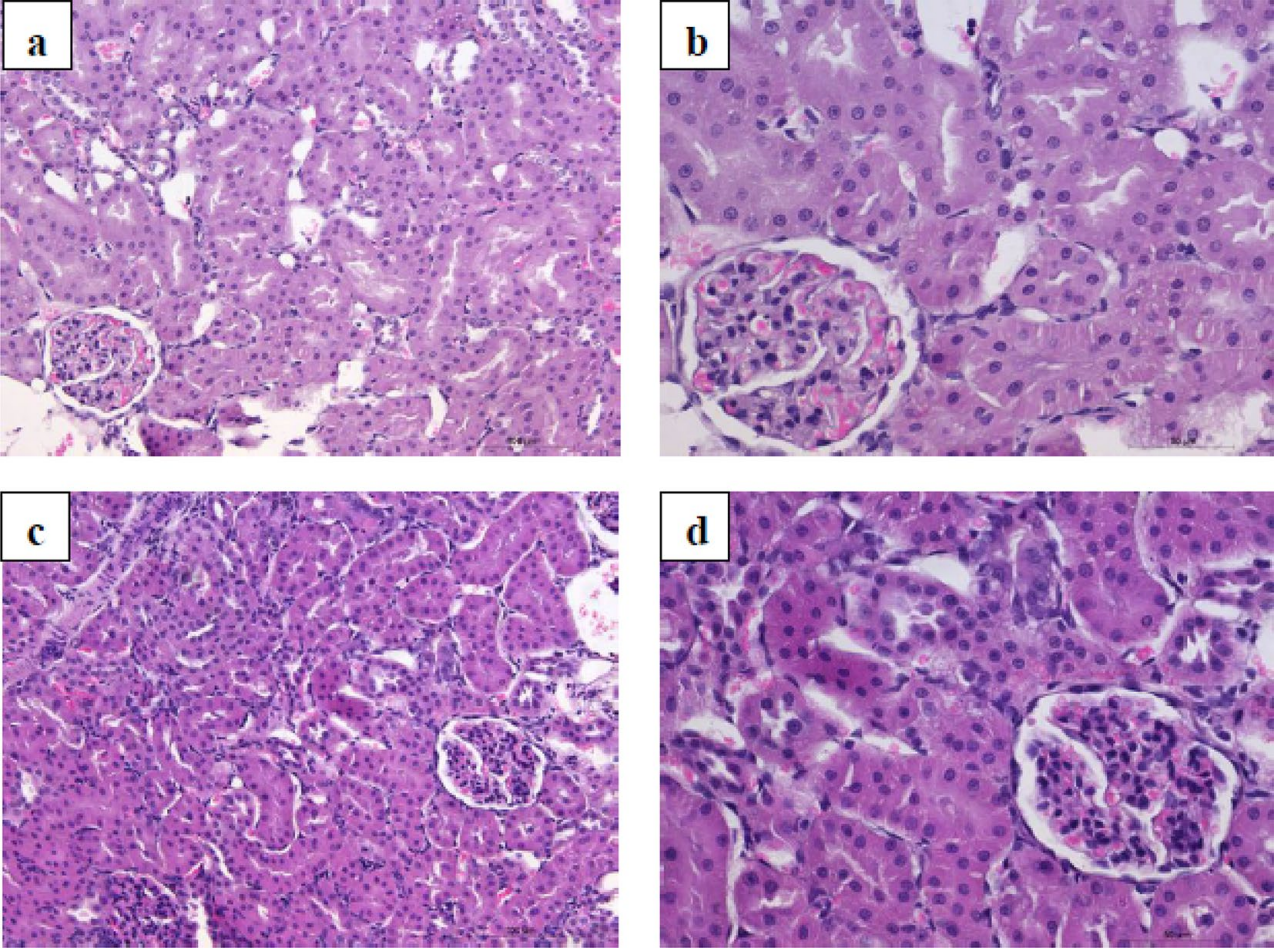
Photomicrographs of HE-stained rat kidney sections. Tissues were stained with haematoxylin and eosin, and image was captured under light microscope with × 20 (**a**,**c**) and × 40 (**b**,**d**) magnifications; (**a**,**b**) tissues from control rats; (**c**,**d**) tissues from rats treated with 1/10 MTD of WD-POM (200 mg per kg). No difference between the kidney tissues from the treated and control rats.

The photomicrographs of the HE-stained kidney tissues excised from the untreated (control) and treated rats are presented in Fig. 6. The sections obtained from the animals treated with 1/10 MTD of WD-POM were similar to those obtained from control rats, showing the kidney section with normal histology (Fig. 6c,d).

**Figure 7:**
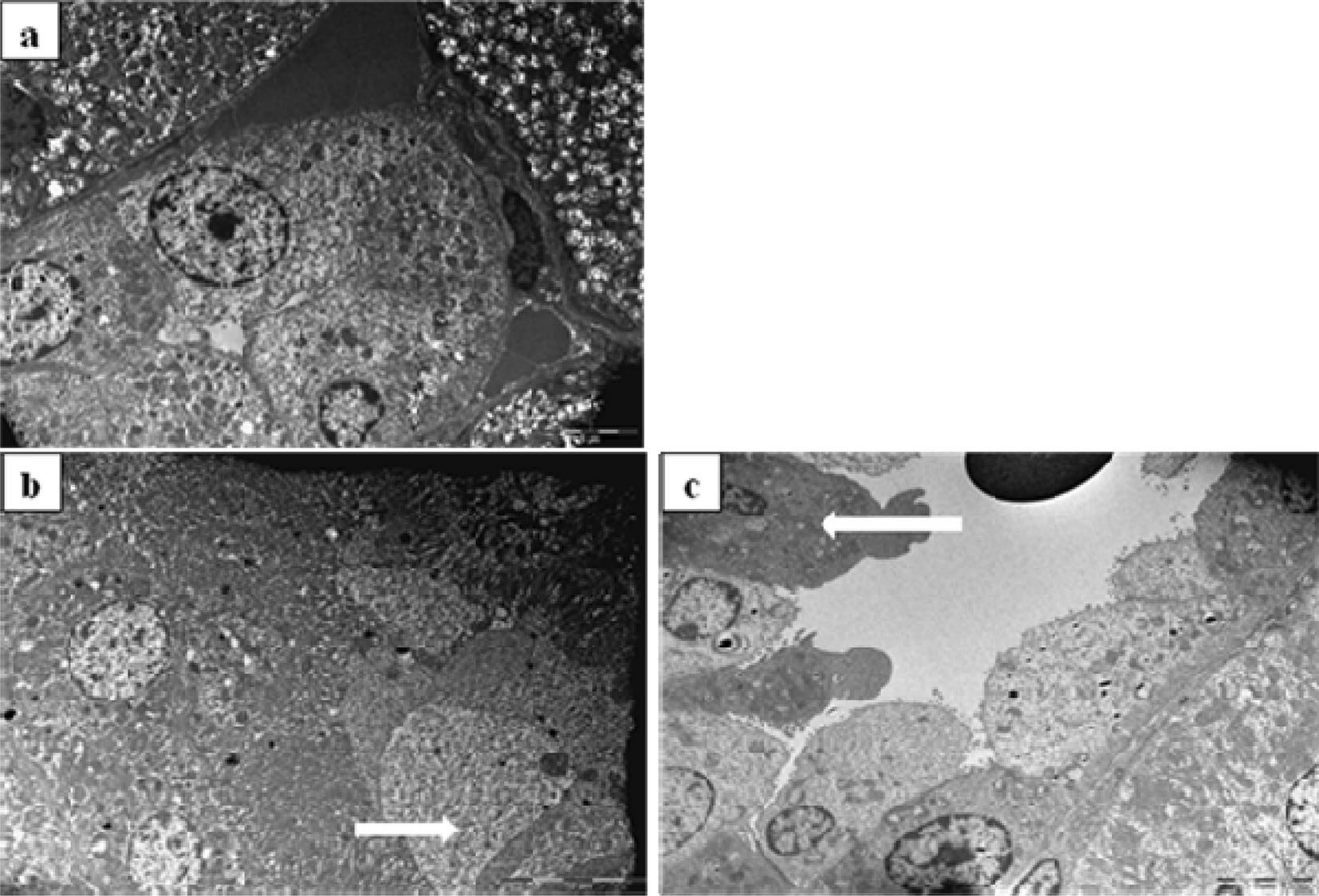
TEM micrographs presenting the ultrastructure of tubules in examined kidneys in: (**a**) (× 2 800) control rat showing unaltered tubular cells; (**b**,**c**) rats treated with 1/10 MTD of WD-POM (200 mg per kg) showing: (**b**) (× 2 800) necrotic cells in tubules (arrow) and (**c**) (× 2 200) apoptotic cells in collecting ducts (arrow).

However, the use of TEM on kidney tissue samples obtained from animals treated with 1/10 MTD of WD-POM revealed the presence of necrosis of tubular cells (Fig. 7b), as well as apoptotic cells in collecting ducts (Fig. 7c).

### Histopathological evaluation of WD-POM—induced liver toxicity

**Figure 8:**
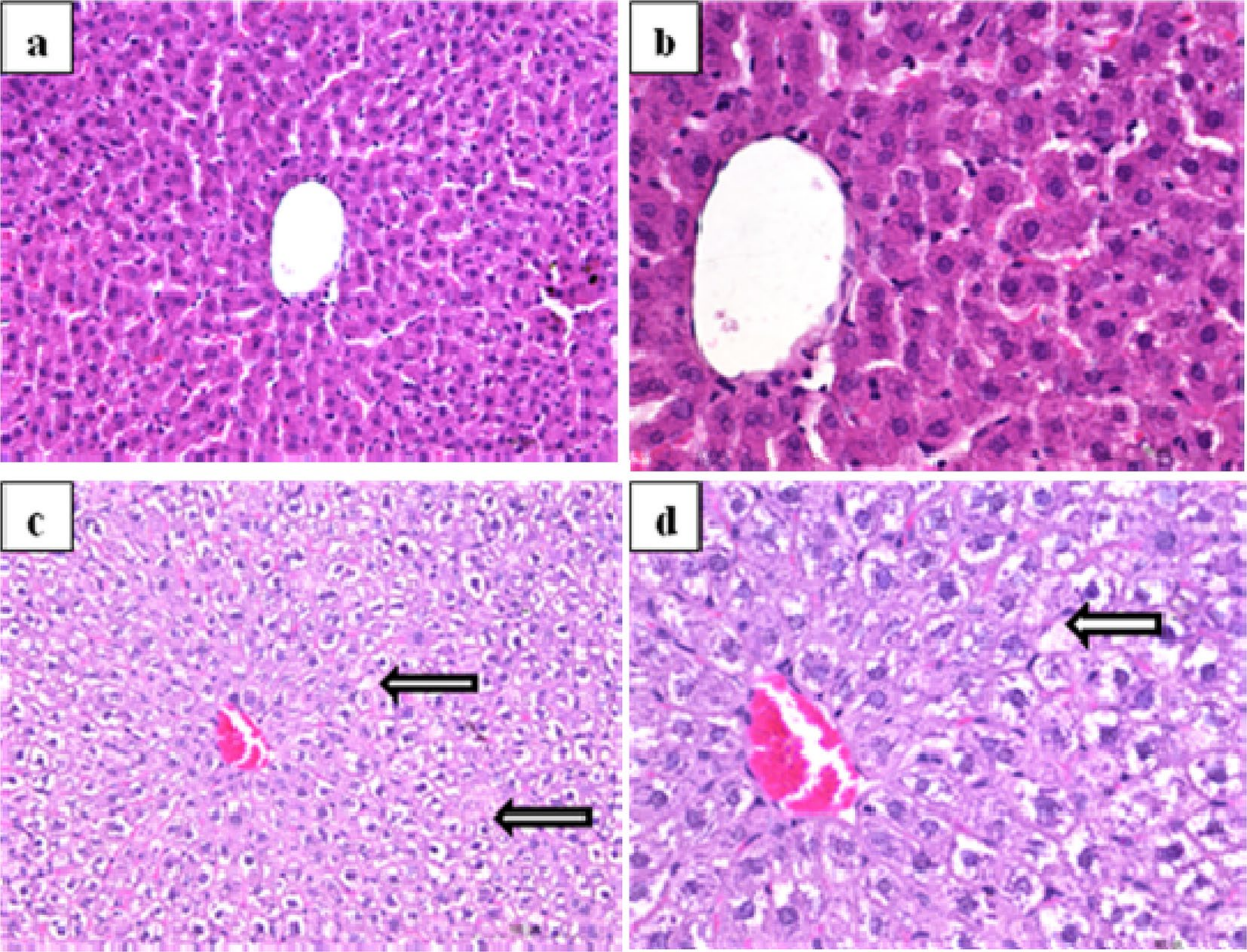
Photomicrographs of HE-stained rat liver sections. Tissues were stained with haematoxylin and eosin, and image was captured under light microscope with × 20 (**a**,**c**) and × 40 (**b**,**d**) magnifications; (**a**,**b**) tissues from control rats showing no irregularities in liver sections; (**c**,**d**) tissues from rats treated with 1/10 MTD of WD-POM (200 mg per kg) showing a vacuolar degeneration of hepatocytes and small areas of necrosis (arrows).

The liver tissue samples taken from animals treated with 1/10 MTD of WD-POM showed a vacuolar degeneration of hepatocytes and discrete small areas of necrosis (Fig. 8c,d).

**Figure 9:**
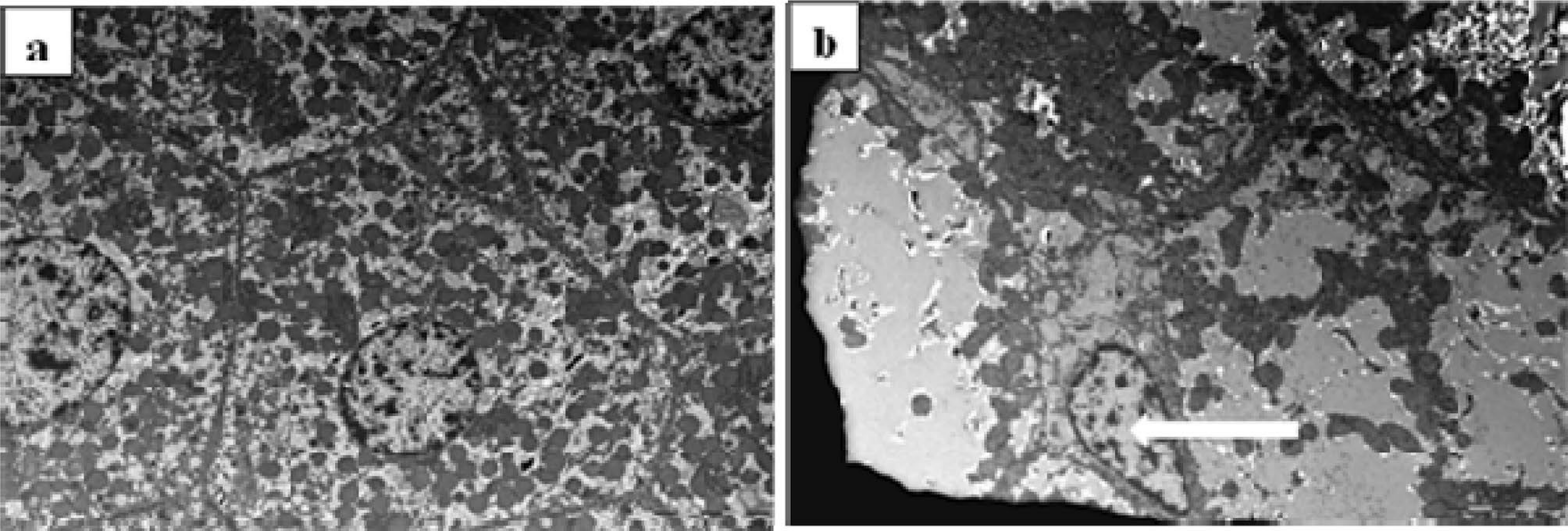
TEM micrographs presenting the ultrastructure of liver (× 2 800). (**a**) Ultrastructural features of unaltered hepatocytes in control rat. (**b**) Liver tissue from rat treated with 1/10 MTD of WD-POM (200 mg per kg) showing a vacuolisation of hepatocytes and necrotic cells (arrow).

The use of TEM on liver tissue samples confirmed the vacuolization of hepatocytes, as well as the presence of necrotic cells (Fig. 9b).

### In vivo biodistribution study of WD-POM

**Figure 10:**
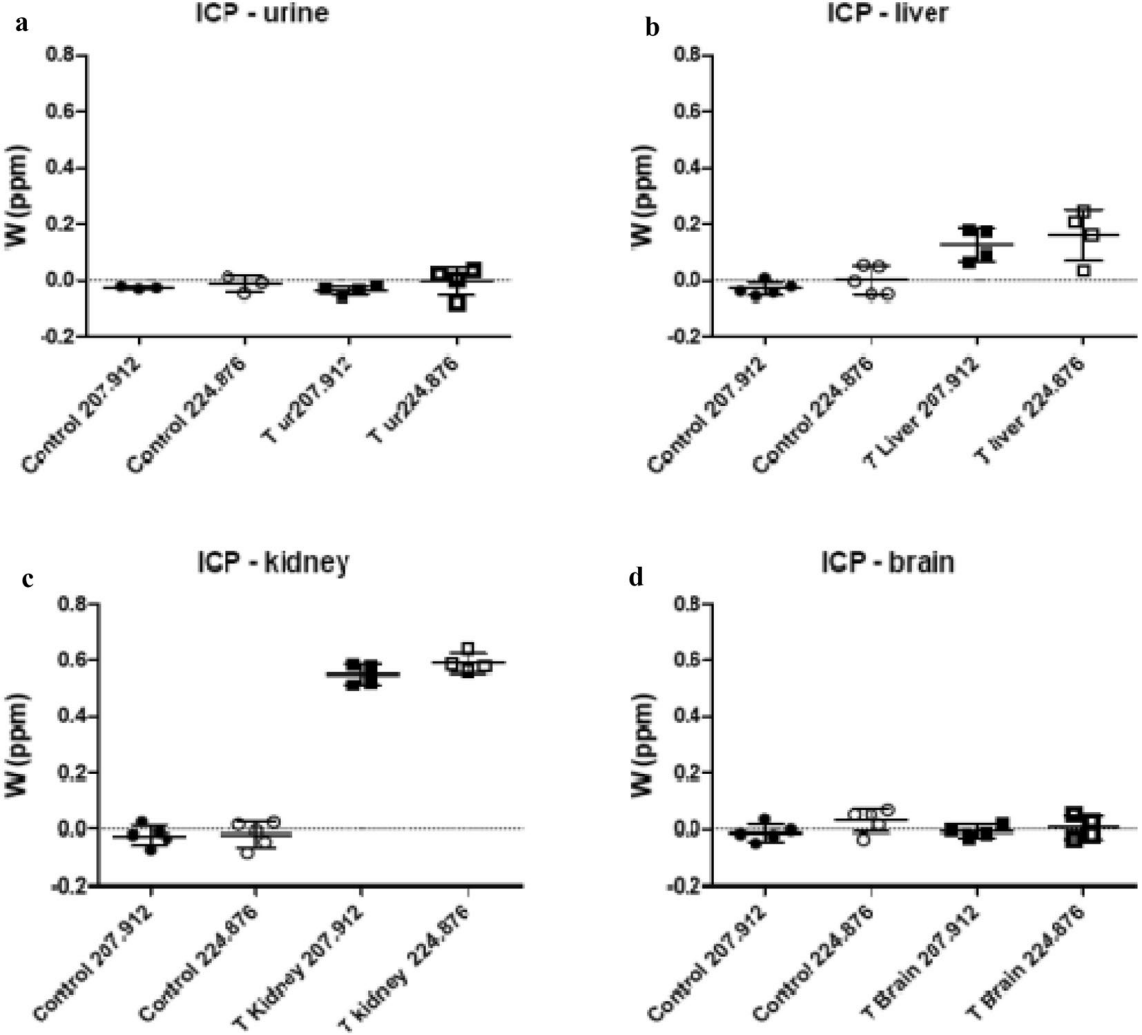
Tissue distribution in *Wistar* rats, expressed as tungsten concentration in: urine (**a**), liver (**b**), kidney (**c**), and brain (**d**), 2 weeks after intravenously administered 1/10 MTD of WD-POM (mean ± SD).

To identify the in vivo biodistribution of WD-POM in animals treated with 1/10 of MTD (200 mg/kg), two weeks after intravenous administration, the content of tungsten in the brain, kidney, liver, and urine samples were measured using ICP-OES. As shown in Fig. 10. the highest deposition was found in the kidney tissue (Fig. 10c), followed by the liver (Fig. 10b). The tungsten concentration of 0.6 ppm was found in the kidney, indicating that this is the major organ for deposition of WD-POM. Approximately four times lower deposition of WD-POM was found in the liver, whereas the presence of tungsten in brain was not detected, suggesting that WD-POM cannot go through the blood brain barrier easily. Interestingly, there was no observed W concentration in urine samples taken from both control and 1/10 MTD group (Fig. 10a).

## Discussion

The purpose of this study was to perform an in vivo toxicity evaluation of Wells–Dawson polyoxotungstate, WD-POM, which previously showed a promising potential as new X-ray contrast agent for CT^12^. Since the WD-POM had never been tested in vivo, the selection of doses and times for monitoring toxicologically relevant parameters was made according to the toxicological recommendations^19–21^. It should be emphasized that the tested WD-POM doses (in mmol W kg^−1^) of 2.474 (1/3 MTD group), 1.485 (1/5 MTD), and 0.742 (1/10 MTD) were significantly higher than the typical injection dose (0.015 mmol W kg^−1^) of tungsten-containing contrast agents used to acquire CT images in vivo^22,23^. Since our results showed the dose-dependent toxicity of WD-POM, toxic effects are expected to be milder in the application of effective doses for clinical application (CT imaging), which are at least fifty times lower in relation to the doses used for the toxicity studies. In addition, the reported promising contrast properties of WD-POM^12^ were confirmed by in vitro CT imaging, which were compared with the standard iodine CT contrast agent, iohexol (Omnipaque™). The obtained apparently higher X-ray attenuation values of WD-POM for the same W/I concentrations (Fig. 1) are in agreement with recently reported contrast performances of tungsten-based nanoparticles which were shown to be superior to commercial iodine CT contrast agents^22,23^. The better CT properties in vitro can be ascribed to the higher linear X-ray attenuation coefficient of tungsten due to its higher electron density in respect to iodine^24^. As WD-POM contains a larger amount of metals per nanocluster (18 W atoms per molecule) than iohexol (3 I atoms per molecule), a stronger X-ray attenuation and adequate contrast can be achieved with lower number of moles (injection doses) of WD-POM, which is of crucial importance from the toxicity point of view. It is worth noting that the solution stability of WD-POM and its dynamics in solution at physiological pH have been studied in detail by the Parac-Vogt's group^12^. WD-POM was found to be partially converted into a stable mono-lacunary WD POM which exhibited satisfactory contrast properties as well^12^. For this reason, it is necessary to compare pharmacokinetics and toxicological profiles of WD-POM and its mono-lacunary product in our further studies.

The mortality rate in the present study was unexpectedly high, especially considering that in similar studies 1/3, 1/5, and 1/10 of MTD did not show such high mortality rates^20,21^. Considering that these doses (1/3, 1/5, and 1/10 of MTD) are generally used for acute intravenous toxicity studies, the most probable explanation for the observed high mortality rate is a high MTD value (2000 mg/kg). Since the MTD was determined after oral administration, one of the explanations could be the inactivation of WD-POM by the low pH of the gastric juice. Another explanation is that due to efflux pumps WD-POM was not appropriately absorbed after oral administration, and thus systematic toxic effects were not observed. Additionally, the compound could be partly inactivated during the process of absorption by the enzymes in the wall of the gastric tube, or it could be affected by the first-pass metabolism^25^. These assumptions are in agreement with a previously published pharmacokinetics study reporting that the oral bioavailability of a POM-based promising drug, Cs_2_K_4_Na[SiW_9_Nb_3_O_40_], in rats is low^26^. Moreover, in our previous toxicological studies on different polyoxotungstates, mild toxic effects were found after oral administration^27,28^, which could be ascribed to the low oral bioavailability of POMs. Additionally, the low oral bioavailability indicates that WD-POM and its related compounds could be a good platform for the development of a new generation of POM-based oral CT contrast agents and suggests the need for further study of absorption and elimination after oral administration.

The obtained results of arterial blood gas analysis indicate that both groups of animals (control and 1/10 MTD group) are in acidosis. There is no significant difference in the pH level between the groups, although there is in the values of *p*CO_2_, HCO_3_^−^, base excess of blood, and anion gap (Table 1). Arterial blood gas analyses are generally interpreted as a whole, taking into account all parameters, and it should not just be looked at as a statistical difference between these two groups. In this manner, we can say that in the control group, the *p*CO_2_ is significantly lower than in the 1/10 MTD group, suggesting that the respiratory system of the control group is attempting to compensate, while in 1/10 MTD is failing^29^. It also means that in the control group most probable reason for low pH is metabolic acidosis^29,30^. The values of base excess in the control group, which are under the lower limit of normal range, confirm the metabolic acidosis in the control group^29,30^. The higher values of the anion gap in the control group, which are also above the normal range, additionally validate the presence of metabolic acidosis^29,30^. In the 1/10 MTD group, low pH, high *p*CO_2_, and the values of base excess that are below the lower limit of the normal range indicate the presence of mixed respiratory and metabolic acidosis^30^. The higher HCO_3_^−^ in the 1/10 MTD group than in the control, p < 0.01 (Table 1) means that the respiratory component of mixed acidosis persists for a longer period^31^.

The use of CO-oximetry is usually indicated for the diagnosis of possible intoxication. CO-oximetry measurement showed a statistically significant increase in the deoxyhemoglobin values and a decrease in oxygen saturation in the 1/10 MTD group compared to the control group (Table 2). Both parameters are also beyond the normal range. Additionally, oxyhemoglobin is lower in the 1/10 MTD group than in the control group. The difference is not statistically significant, but oxyhemoglobin values are below the normal range in this group. The deoxyhemoglobin is the hemoglobin without oxygen and is usually increased in hypoxia. Any disorder that causes hypoxia, may increase deoxyhemoglobin level^31^. The oxyhemoglobin presents the amount of hemoglobin saturated with the oxygen and low values of this hemoglobin mean that the animals did not get enough oxygen. The obtained CO-oximetry values suggest that intravenous application of WD-POM (200 mg per kg) could harm the animal lungs, which can explain the presence of mixed acidosis. The assumption is in agreement with previously reported tungsten-associated lung pathologies^32,33^ and suggests the need for further detailed toxicological examinations.

Glucose levels were elevated in both groups (control and 1/10 MTD group) without significant differences between the groups. The rise in the glucose level is a consequence of surgical intervention performed to expose the heart for blood sampling. The stress induced-hyperglycemia is a common and expected response to anesthesia and surgical interventions^34–36^. The lactate levels were also increased without statistically significant differences between the groups. Similar to the rise of glucose, the stress-induced rise of lactate levels is also common in anesthesia and surgical interventions^37–39^. This is known as the B type of lactic acidosis and is a consequence of extreme acceleration in metabolism owing to stress^38^. The high lactate level is the cause of isolated metabolic acidosis in the control group and metabolic part of mixed respiratory and metabolic acidosis in 1/10 MTD group. The observed higher values of lactate in the control group could be the explanation for higher anion gap values in this group since the anion gap values vary depending on lactate levels^30^.

The electrolyte concentrations measured in this study (Na^+^, K^+^, Cl^−^ Ca^2+^, Mg^2+^, Ca^2+^, and Mg^2+^ normalized to pH 7.4) were not significantly statistically different between the two groups (Table 3). The only exception is potassium levels which are significantly lower in the 1/10 MTD group.

Since the kidney is the major excretory organ for most substances, the combination of functional and histological parameters was monitored to evaluate the nephrotoxic properties of WD-POM. The renal function parameters (creatinine and BUN levels) determined two weeks after the intravenous application of WD-POM (200 mg per kg) were within the physiological range and did not show statistically significant alterations concerning the control group (Fig. 5). In addition to these parameters, the obtained values for electrolyte concentrations indicate that WD-POM at the applied dose did not significantly disturb the function of the kidneys (Table 3), which are primarily responsible for the regulation of fluid and electrolyte balance. On the contrary, despite the fact that light microscopy did not indicate differences between kidney samples taken from the control and 1/10 MTD group (Fig. 6), the morphological studies by TEM (Fig. 7) showed the presence of necrosis of tubular cells as well as apoptotic cells in collecting ducts. These nephrotoxic findings for WD-POM could be explained by the obtained highest accumulation of tungsten in the kidneys (Fig. 10c). The presented results of in vivo biodistribution of WD-POM in animals treated with 1/10 of MTD (200 mg/kg) (Fig. 10) are consistent with a previously published the highest deposition of tungsten in *Wistar* rat kidneys 24 and 48 h after 180 mg/kg of orally administered polyoxotungstate, Cs_2_K_4_Na[SiW_9_Nb_3_O_40_]^26^. Although the concentration of tungsten determined in the liver tissue samples was approximately four times lower than in the kidney tissue, the results of the histological analysis revealed morphological irregularities in liver sections from rats treated with 1/10 MTD of WD-POM (Figs. 8 and 9). These findings are consistent with our previously published studies regarding in vivo toxicity evaluation of POM-based compounds, suggesting that the kidney and liver are the vital positions of POM metabolism and elimination process^27,28^.

In conclusion, we report the first in vivo toxicological study of a nano-sized water soluble cluster with promising potential as a CT contrast agent. We have shown that Wells Dawson POM exhibits dose dependent toxicity, proving that lung, kidney, and liver are the most likely toxic sites. However, it needs to be pointed out that the investigated WD-POM doses used in this work were based on the toxicological recommendations, which are at least fifty times higher than the typical injection dose (0.015 mmol W kg^−1^) of tungsten-based contrast agents that are used to acquire CT images in vivo. Therefore, the potential clinical application would require further toxicological study involving exposure to lower doses of WD-POM and its related compounds, which are sufficient for in vivo CT imaging. This study is the first and important step in evaluating toxic effects of POM based nanoclusters, which in recent years have also shown a large potential as inorganic drugs against virus, bacteria, and tumor cells.

## Methods

### Chemicals

Parent Wells–Dawson POM, α-/β-K_6_P_2_W_18_O_62_ (WD-POM) was synthesized as described in the literature^40^, and dissolved in a saline solution. WD-POM stock solution (440 mg/mL) was prepared by vigorous mixing at 50 °C and used for MTD determination. For the administration of 1/3, 1/5, and 1/10 MTD the stock solution was diluted by saline up to desired concentrations immediately before use.

### In vitro CT imaging

The in vitro X-ray phantom images of WD-POM solutions (W concentrations (in mM): 100, 50, 25, 12.5, 6.25, and 3.125) were acquired in plastic test tubes using a clinical CT GE Medical Systems Revolution Evo 128-Detector CT scanner (parameters used were: the x-ray source current = 400 mA, the X-ray source voltage = 80 kV). In addition, phantom images of commercial iodine CT contrast agent, iohexol solutions, C_19_H_26_I_3_N_3_O_9_ (Omnipaque™, 350 mg I/mL) (I concentrations (in mM): 100, 50, 25, 12.5, 6.25, and 3.125) were acquired under the same conditions. Water served as a reference for Hounsfield units (HU) of 0.0.

### Experimental animals and ethical approval

Experiments were performed on healthy, male, 5 weeks old *Wistar albino* rats weighing 180–220 g. The experimental animals were housed in carbon cages (3–4 per cage) and had ad libitum access to standard rodent pellet food and tap water. Environmental conditions were maintained constant during the whole study period with a 12 h/12 h dark/light cycle, at 23 ± 2 °C, and humidity 55 ± 10%.

The methodological procedures performed in this study were approved by Ethical Commission for Experimental Animal Welfare Protection (Faculty of Medicine, University of Belgrade, N°6447/1-2020). All procedures were done following Guidelines from the European Convention for the Protection of Vertebrate Animals Used for Experimental and other Scientific Purposes, and the Good Laboratory Practice (GLP) was fully applied. The study is reported in accordance with ARRIVE (Animal Research: Reporting of In Vivo Experiments) guidelines^41^.

### Experimental design of single-dose intravenous acute study

In the preliminary sighting study, the MTD for WD-POM (2000 mg/kg) was determined after oral administration. The MTD is a dose that produces some signs of toxicity without causing severe toxic effects or mortality. The OECD/OCDE guidelines for Acute Oral Toxicity–Fixed dose procedure^42^, and the Guidance Document on the Recognition, Assessment and Use of Clinical Signs as Humane Endpoints for Experimental Animals Used in Safety Evaluation^43^ were used for determining MTD. Based on the established MTD the experimental groups were created in the following step^19–21^. Twenty male *Wistar* rats were randomly distributed into one of the four groups: (1) control, (2) 1/3 MTD, (3) 1/5 MTD, and (4) 1/10 MTD, which received intravenously 666.7 mg/kg (1/3 MTD group), 400 mg/kg (1/5 MTD group), and 200 mg/kg (1/10 MTD group), while the control group received saline solution. To comply with the 3R rule (replacement, reduction, and refinement of laboratory animals) the exact number of animals was calculated before the beginning of the study^44^. The calculation of sample size was done by power analysis (effect size and standard deviation were taken from our previous study^27^, type I error was set at 5%, power was 80%, the two-tailed test was used, and expected attrition of sample size was set at 20%), and then controlled with resource equation method.

**Figure 11:**
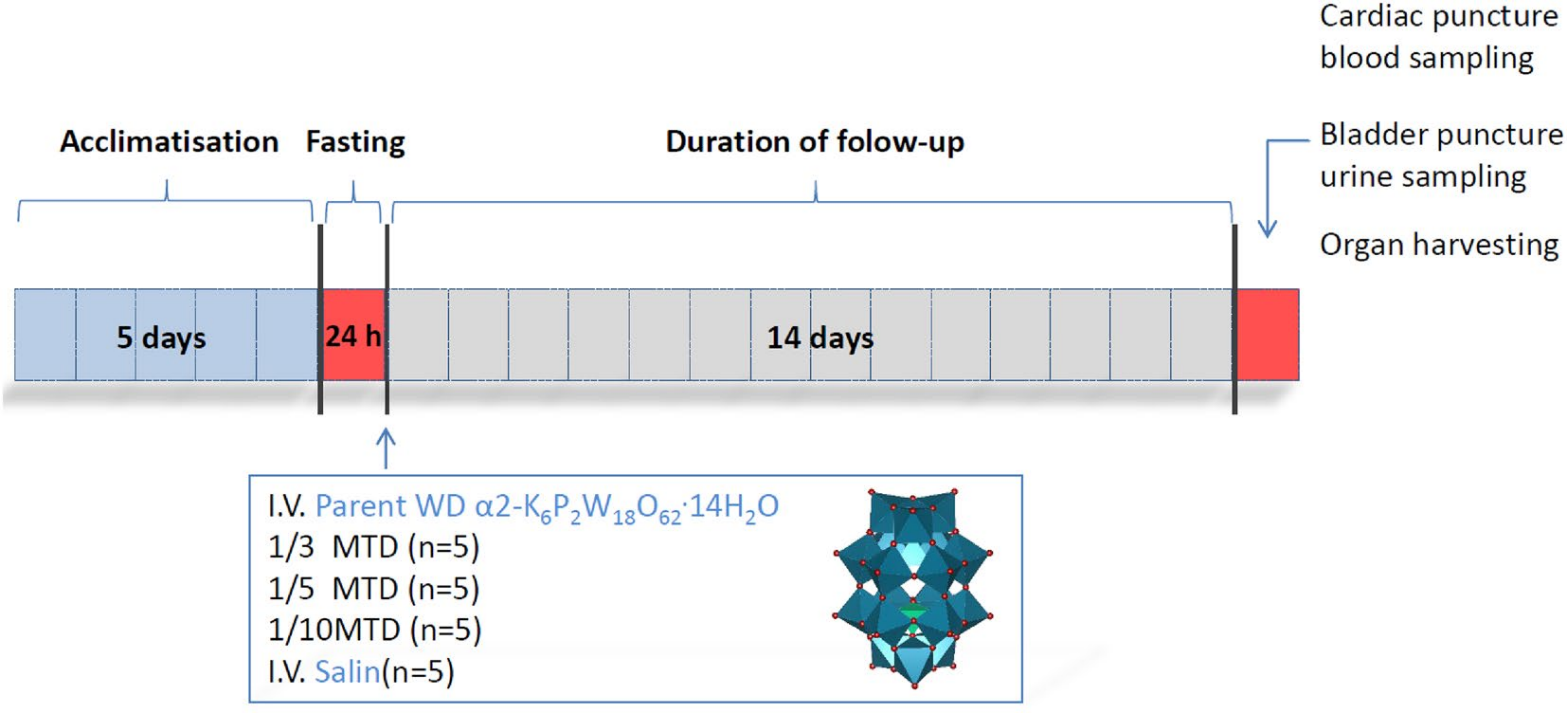
The experimental design for the in vivo acute toxicity evaluation of WD-POM.

Before the experiments were started, the animals were allowed five days acclimatization period to laboratory housing conditions after they have been brought from the central housing facility. The food was withdrawn overnight before the WD-POM application. The different WD-POM concentrations, as well as saline solution for the control group, were administrated intravenously in the lateral tail vein. The volume of administration did not exceed 1 mL. The intravenous route was chosen due to the possible use of WD-POM as a contrast agent. Immediately after the administration of WD-POM or saline solution the food was retrieved. Twice daily for the next 14 days animals were observed closely for mortality and any notable behavioral or physical (i.e. skin, visible mucosa, and fur) changes, as well as for visible symptoms of toxicity. The experimental design is summarized in Fig. 11.

### Blood and urine sampling and organ collection

At the end of the follow-up period, the animals were weighed to calculate the exact dose of urethane for the anesthesia. The urethane was freshly prepared on the day of the experiment and was administrated as a single intraperitoneal injection in a dose of 125 mg per 100 g of animal body weight. After urethane administration, the animals were allowed 90 min for stabilization, after which the depth of anesthesia was determined. If animals did not respond to tail stimulation, when the tail tip was poked with a needle, the anesthesia was considered superficial surgical anesthesia. The cessation of pedal withdrawal effect, when the space between tows was pinched with tweezers, was considered as a state of deep surgical anesthesia.

After entering the deep surgical anesthesia animal's abdomen and chest were open, which exposed the animal's heart. The heparinized syringe for arterial blood gas analysis was used to obtain blood samples from the exposed heart. To obtain arterial blood the needle of the syringe was placed in the left heart. The opening of the animal chest and exposing of the heart were done to ensure arterial blood collection. The syringe was thoroughly mixed after blood was collected to prevent clothing. Post blood sampling, the animals were decapitated and the whole brain, liver, and kidneys were harvested from each animal. The urine samples were collected by bleeder punction with regular (unheparinized) syringe and needles. The liver and kidney samples for light and electron microscopy were fixed in 4% buffered formaldehyde and 3% glutaraldehyde, respectively. The urine and tissue samples of the brain, kidney, and liver for biodistribution study were kept at − 80 °C.

### Laboratory blood analysis

Immediately after the blood was collected, blood gas analysis (pH, pCO_2_, HCO_3_^−^, base excess of blood, anion gap), CO-oximetry parameters (total hemoglobin, oxyhemoglobin, carboxyhemoglobin, methemoglobin, deoxyhemoglobin, oxygen saturation, oxygen content of hemoglobin and oxygen capacity of hemoglobin), electrolytes (Na^+^, K^+^, Cl^−^, Ca^2+^, Mg^2+^, ionized calcium and magnesium normalized to pH 7.4), glucose, lactate, creatinine, and blood urea nitrogen (BUN) levels were measured with Profile Prime Plus® Critical Care Analyzer (Nova Biomedical Co., Waltham, Massachusetts, USA).

### Histological evaluation

The tissue samples for light microscopy were dehydrated and fixed with paraffin. The tissue in paraffin was cut into 5 µm tick sections and stained with Hematoxylin and Eosin (HE). A light microscope (Leica DMLS) with a digital camera (Leica DFC295) was used to take micrographs.

For electronic microscopy, the tissue samples, previously fixed with 3% glutaraldehyde in cacodylate buffer, were postfixed in 1% OsO_4_. Following dehydration in graded alcohols, the cells were fixed in an Epoxy medium (Sigma-Aldrich, 45345). Thin sections were mounted on copper grids (Sigma-Aldrich, G4901), and stained with uranyl acetate and lead citrate for examination on an electron microscope (Morgagni 268D, FEI, Hillsboro, OR).

### In vivo biodistribution study

0.1 g of the tissue sample (brain, kidney, liver), 5 mL HCl, 4 mL HNO_3_ and 2 mL HBF_4_ were added to a Teflon vessel. Vessels were left open and gently shaken for 5 min before being sealed. Samples were digested using the pre-set ‘Easy Prep *i*wave Zeolite’ protocol on a MARS6 microwave (15 min ramp time to 210 °C, 15 min hold at 210 °C, 30 min cool down before opening of vessel). After cooling, each acid digested mixture was diluted to 2% HNO_3_ using ultra-pure water (Milli-Q) and made up to a final volume of 10 mL as analyte. 0.1 mL of the urine samples were treated in the same way as tissue samples.

The samples were analyzed directly by Inductively coupled plasma-optical emission spectrometry **(**ICP-OES), using PerkinElmer optical emission spectrometry Avio 500 instrument. Tungsten concentrations were calculated using the calibration curve, which was profiled with a series of standard tungsten solutions of different concentrations (0.0001–50 ppm).

### Statistical analysis and data presentation

The statistical analysis was performed within Graph Pad Prism (Graph Pad Software, San Diego, California, USA). Survival graphs were constructed according to the Kaplan–Meier method with a Long-rank test used for curve comparison. The difference between the two groups was determined using *Student's two-tailed t-*test. The results are presented as mean ± standard deviation (SD). The obtained values were significant if the p values were below 0.05.

## Data Availability

The datasets used and/or analyzed during the current study are available from the corresponding author upon reasonable request.
